# Differential effects of static and dynamic visual prompts on attention–motor performance: the role of sustained attention after stroke

**DOI:** 10.3389/fneur.2026.1845909

**Published:** 2026-09-18

**Authors:** Yi Zhang, Weinv Fan, Yahui Li, Jie Zhang, Guohong Chai, Min Tang, Lanxiang Wang, Jialin Xu

**Affiliations:** 1 Ningbo Institute of Materials Technology and Engineering, Chinese Academy of Sciences, Ningbo, Zhejiang, China; 2 Department of Neurology, Ningbo No. 2 Hospital, Ningbo, Zhejiang, China; 3 Innovation Centre for Information, Binjiang Institute of Zhejiang University, Hangzhou, China; 4 Department of Neurological Rehabilitation, Ningbo Rehabilitation Hospital, Ningbo, Zhejiang, China; 5 Rehabilitation Medicine Department of the 8th Medical Center of Chinese PLA General Hospital, Beijing, China

**Keywords:** attention-motor integration, brain functional network, functional near-infrared spectroscopy, stroke, visual prompt

## Abstract

**Introduction:**

Visual prompts enhance motor performance in goal-directed tasks by modulating attentional resource allocation. Given that stroke patients often exhibit attentional deficits and impaired visual processing, cue-based interventions are increasingly used in neurorehabilitation. However, how different prompt types interact with attentional capacity to influence attention–motor performance remains unclear. This study aimed to examine the differential effects of static and dynamic prompts in stroke patients and healthy controls.

**Methods:**

Sustained attention was assessed using the Sustained Attention to Response Task (SART) in stroke patients and healthy controls. A virtual reality-based Target-Goal Task (TGT) was employed to compare the effects of static and dynamic prompts between groups.

**Results:**

Stroke patients showed significant impairments in attention–motor performance compared with controls. In healthy individuals, dynamic prompts were associated with faster response times, higher prefrontal oxygenated hemoglobin levels, and more efficient brain network topology (i.e., higher clustering coefficient [CC] and shorter characteristic path length [PL]), indicating enhanced attentional allocation and information integration. In contrast, stroke patients performed better under static cue conditions, possibly due to lower cognitive load demands.

**Discussion:**

The effects of visual prompts depend on sustained attention capacity. These findings highlight the importance of tailoring prompt strategies to individual cognitive profiles and provide evidence for personalized visual cue-based rehabilitation.

## 1 Introduction

In goal-directed tasks, stroke patients can effectively improve training efficiency and promote motor function recovery by actively engaging in the formation and execution of motor intentions. As an important external regulatory approach, visual prompts play a critical role in neurorehabilitation by guiding attentional allocation and facilitating motor planning (1, 2). Previous studies have shown that providing visual prompts prior to target onset helps individuals allocate attentional resources in advance and optimize motor preparation, thereby improving task performance (3–5). However, stroke patients commonly exhibit attentional deficits and reduced efficiency in visual information processing, which limits their ability to process and utilize complex cue information (6–8). Therefore, designing visual prompts that are adapted to individuals with limited attentional resources, in order to enhance visual–motor integration efficiency in goal-directed tasks, has become an important issue in current rehabilitation research.

In the field of visual prompt research, the cue–target paradigm has been widely adopted, with its core feature being the presentation of predictive visual prompts prior to target onset (7, 9). Among these, static cue prompts represent the most common form, typically indicating the upcoming target location and guiding individuals to allocate attentional resources to a specific spatial region (10, 11). For example, in the classical Posner cueing paradigm, presenting a simple box at the target location as a cue can significantly shorten reaction times when the target appears at the cued position (12, 13). From a neural perspective, static cue prompts can modulate brain network activity associated with attentional orienting and motor preparation (14). Electroencephalography studies have shown that valid spatial cues can elicit early components related to attentional orienting (e.g., N2pc) as well as motor preparation–related components such as the lateralized readiness potential (9). Functional magnetic resonance imaging studies further indicate that cue-based prediction of target location modulates activity in attention-related networks, including the intraparietal sulcus and frontal eye fields (15, 16). thereby providing a neural basis for anticipatory allocation of attentional resources (17). Although static cue prompts have clear advantages in guiding spatial attention, they lack temporal dynamic information and may be insufficient to maintain stable attention over prolonged task durations, potentially leading to attentional fatigue or reduced vigilance (18).

In contrast, dynamic properties of visual stimuli (e.g., motion or luminance changes) are considered more salient in capturing attention (19). Sudden changes in the visual environment can rapidly attract attention and improve the efficiency of resource allocation (20, 21), and previous studies have demonstrated that dynamic prompts exert stronger guidance effects than static prompts (22). Based on this, recent research has begun to explore prompt designs that integrate multidimensional information to better approximate complex real-world information processing demands. In the present study, a novel form of visual prompt, termed dynamic combined prompts, is proposed. This type of prompt presents a dynamic visual stimulus at the target location in advance (e.g., gradually disappearing in a clockwise manner), providing not only spatial predictive information but also temporal information about target onset through its dynamic changes, thereby enabling coordinated modulation of attentional allocation and motor preparation. Previous studies have shown that multidimensional dynamic information can more effectively capture and sustain attention (23), promote activation of brain networks associated with attention and motor integration (24), and enhance interregional information integration during task performance (25). However, the effectiveness of dynamic prompts is task-dependent. Under conditions of high cognitive load, more complex prompts may reduce usability due to increased processing demands. This issue is particularly critical for stroke patients: given that they may have limited attention–motor integration capacity, the processing demands imposed by the multidimensional information embedded in dynamic combined prompts may exceed their available cognitive resources, thereby increasing cognitive load and exacerbating attentional fatigue (26, 27). Therefore, the design of visual prompts in stroke rehabilitation should balance attentional capture and cognitive load (28, 29).

Based on the above theoretical and empirical background, this study established two types of visual prompt conditions: static cue prompts and dynamic combined prompts. Dynamic combined prompts provide spatial information while simultaneously conveying temporal expectations of target onset through a gradual disappearance process, thereby facilitating motor preparation. In contrast, static cue prompts provide concise spatial information, reducing visual interference and enhancing attentional stability. Using young healthy individuals as a control group, this study systematically compared attention–motor integration performance between stroke patients and healthy individuals under the two prompt conditions by combining behavioral measures, hemodynamic indicators, and graph-theoretical metrics of brain functional networks. The aim of this study was to preliminarily elucidate the mechanisms by which different visual prompts influence attention–motor performance and to provide theoretical and empirical support for optimizing individualized visual prompt–based interventions in stroke neurorehabilitation.

## 2 Materials and methods

### 2.1 Participants

A total of 14 stroke patients and 12 healthy controls were recruited for this study. Stroke patients were recruited from Ningbo Rehabilitation Hospital, whereas healthy controls were recruited from nearby communities and universities. The sample size was determined primarily based on the exploratory nature of the study, the feasibility of participant recruitment, and sample sizes used in previous fNIRS studies involving stroke patients (30, 31). Two stroke patients were excluded because excessive head movement during the experiment resulted in severe motion artifacts and failure to meet the predefined fNIRS data-quality criteria. Therefore, 12 stroke patients and 12 healthy controls were included in the final analysis.

The inclusion criteria for the stroke group were as follows: (a) a first-ever unilateral supratentorial stroke, including ischemic or hemorrhagic stroke, confirmed by cranial CT or MRI; the side of hemiplegia was not considered an inclusion criterion; (b) ≥ 6 months since stroke onset and a clinically stable condition; (c) age between 40 and 85 years; (d) unilateral upper-limb motor impairment following stroke, with sufficient motor function of the non-paretic upper limb to perform the required key-pressing and operational tasks; participants’ ability to correctly perform the required operations was confirmed through standardized task instructions and practice before the formal experiment; (e) essentially preserved cognitive function, with an MMSE score ≥24; (f) no obvious visual field deficits or hemispatial neglect, as confirmed by clinical visual field examination and screening for hemispatial neglect; and (g) clear consciousness and the ability to understand the experimental instructions and complete the tasks independently.

The exclusion criteria for the stroke group were as follows: (a) severe aphasia or cognitive impairment that prevented the participant from understanding or following the experimental instructions; (b) other neurological or psychiatric disorders that could affect brain function or task performance; (c) severe upper-limb pain, joint contracture, orthopedic disorders, or other musculoskeletal conditions that could interfere with task performance; (d) severe visual or auditory impairment that prevented the participant from accurately perceiving the experimental cues; (e) other conditions that could affect fNIRS signal acquisition or experimental safety; and (f) excessive head movement during the experiment resulting in failure to meet the predefined fNIRS data-quality criteria.

The inclusion criteria for the healthy control group were an age of 23–27 years, normal cognitive function as assessed using the MMSE, no history of stroke or other neurological disorders, and the ability to understand the experimental instructions and complete the experimental tasks. The exclusion criteria were the presence of psychiatric disorders, severe visual or auditory impairment, orthopedic conditions affecting upper-limb movement, or other conditions that could affect task performance or fNIRS signal acquisition.

For the stroke group, clinical and screening information was obtained from the hospital information system and electronic medical records, including age, stroke type, lesion location identified by neuroimaging, time since stroke onset, side of hemiplegia, MMSE score, visual field status, hemispatial neglect screening results, and relevant medical history. The records also provided information on aphasia, visual or auditory impairment, upper-limb pain or contracture, and other conditions relevant to task performance, fNIRS signal acquisition, or experimental safety. Task-related upper-limb motor ability and task comprehension were confirmed through standardized task instructions and practice before the formal experiment. Information relevant to the predefined inclusion and exclusion criteria was used for participant screening, whereas stroke type, lesion location, time since stroke onset, side of hemiplegia, and MMSE scores were additionally used to characterize the clinical features of the stroke group. The stroke group had a mean age of 64.7 ± 14.4 years and included 8 males and 4 females; 4 patients had right-sided hemiplegia and 8 had left-sided hemiplegia.

For the healthy control group, demographic and screening information was collected during recruitment and pre-experiment assessment, including age, cognitive function, neurological and psychiatric history, visual and auditory function, orthopedic conditions affecting upper-limb movement, and other conditions that could affect task performance or fNIRS signal acquisition. Task comprehension and the ability to complete the required tasks were also confirmed before the experiment. These data were used to determine eligibility and characterize the basic features of the healthy control group. The healthy control group had a mean age of 25.1 ± 0.9 years and included 10 males and 2 females; all healthy controls were right-handed.

Except for age, which was included as a covariate in the relevant analyses, these clinical and screening variables were not included as independent variables or covariates in the main statistical analyses.

All participants were conscious, clinically stable, and able to complete the experimental tasks under the guidance of the researchers. Written informed consent was obtained from all participants before the experiment. The study was conducted in accordance with the Declaration of Helsinki (2008 revision of the World Medical Association), and the study protocol was approved by the Ethics Committee of Ningbo Rehabilitation Hospital.

### 2.2 Data acquisition

Hemodynamic responses during the TGT were recorded using a Hitachi ETG-4100 functional near-infrared spectroscopy (fNIRS) system. The system was operated at a sampling rate of 100 Hz to ensure high temporal resolution for capturing task-related hemodynamic signals. An optimized 46-channel probe configuration was employed, consisting of 18 near-infrared light emitters and 15 photodetectors. A standard source–detector separation of 30 mm was used to achieve an optimal balance between penetration depth and spatial sampling density. During the experiment, participants wore a calibrated optode cap. All channels were checked for signal quality prior to data acquisition, and channels affected by potential interference (e.g., hair obstruction) were adjusted or excluded to ensure that data quality met established fNIRS research standards.

### 2.3 Experimental procedure

All experiments were conducted in a quiet environment at Ningbo Rehabilitation Hospital. Prior to the experiment, participants were asked about their emotional and physical state. They were seated comfortably in front of a table, with a display screen positioned 75 cm away. Before the formal experiment, the task instructions were explained, followed by practice sessions of approximately 3 min to ensure that participants understood the task requirements and could perform the required operations independently. Throughout the experiment, participants were instructed to minimize unnecessary head movements and swallowing. After each task, participants reported their perceived fatigue and task completion status, and rest periods were provided according to individual needs. To minimize the potential influence of upper-limb motor impairment on task performance, all stroke patients performed the experimental tasks using their non-paretic hand.

The experimental procedure consisted of two tasks: the Sustained Attention to Response Task (SART) and the Target-Guided Task (TGT), separated by a 5-min rest interval to reduce potential fatigue carryover effects. All participants completed the SART first, followed by the TGT. The task order was fixed to avoid potential interference from the relatively complex TGT with the assessment of sustained attention.

The SART employed a classic Go/No-go paradigm to assess sustained attention. A schematic of the task is shown in Figure 1A. The task was programmed and presented using E-Prime 3.0 software. The stimuli consisted of black digits (1–9) randomly presented at the center of a white screen. In each trial, a blank screen was presented for 1,250 ms, followed by a digit stimulus displayed for 250 ms, resulting in a total trial duration of 1,500 ms. Participants were instructed to press the spacebar in response to Go stimuli (digits 1, 2, and 4–9; probability = 88.9%) and to withhold their response to the No-go stimulus (digit 3; probability = 11.1%). The formal SART consisted of one block containing 180 trials. Reaction time, defined as the interval from Go-stimulus onset to the corresponding key press, and response accuracy were recorded. Accuracy included commission errors, defined as responses to the No-go stimulus, and omission errors, defined as failures to respond to Go stimuli.

**Figure 1:**
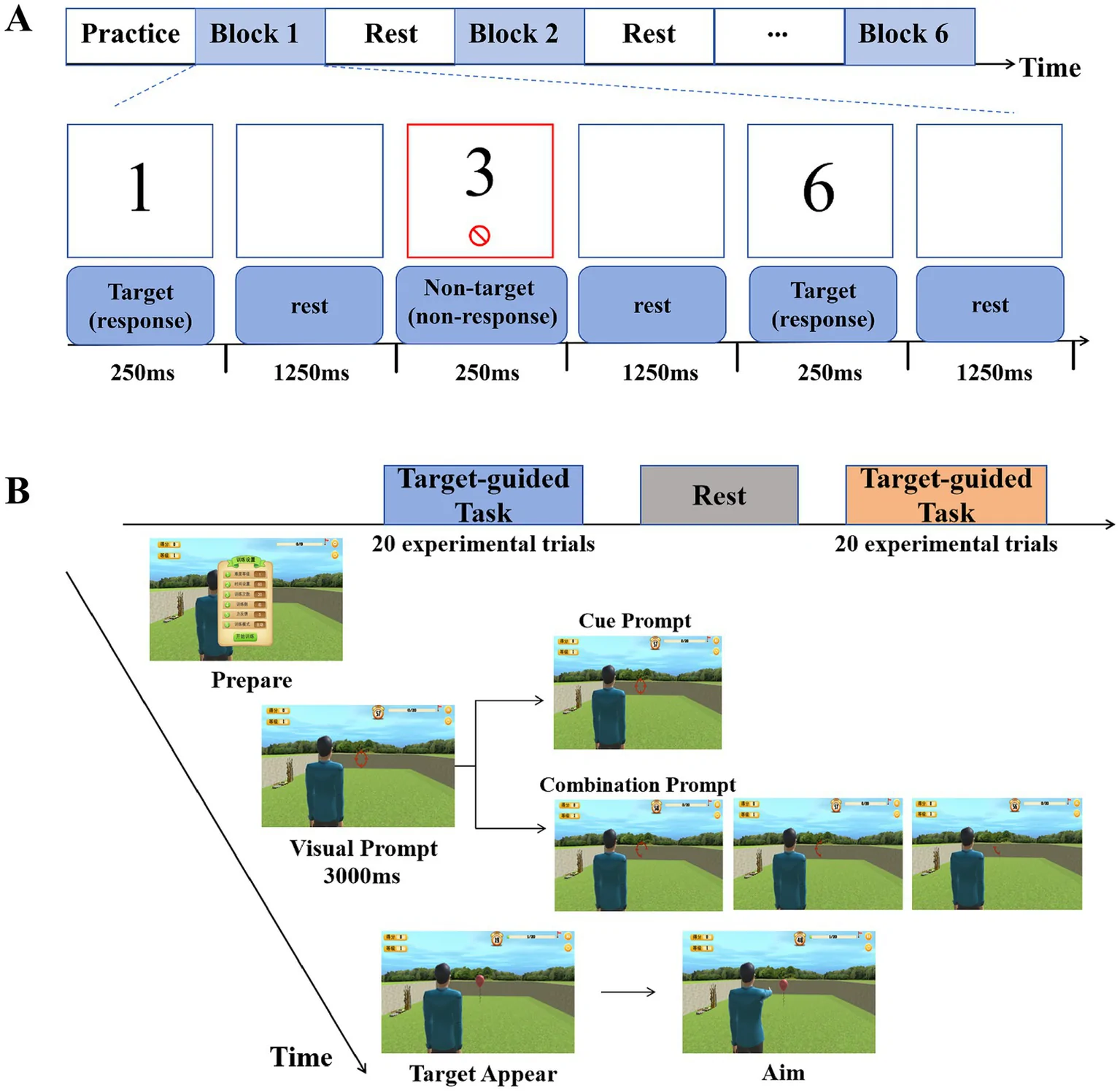
Design diagrams of each experimental paradigm. **(A)** The sustained attention task employs the classic Go/No-go paradigm. Participants are required to respond to high-frequency non-target digits (1, 2, 4–9) by pressing a designated key, while withholding their response to the low-frequency target digit (3). In each trial, the digit stimulus is displayed for 250 ms, followed by a blank screen presented for 1,250 ms. **(B)** The target-guided task experiment employs a paradigm comprising two prompt conditions: static cues and dynamic combined prompts. Each trial includes a 3-s prompt presentation phase followed by a 60-s response window.

The TGT was designed as a first-person virtual shooting scenario, enabling real-time interaction between visual prompts and motor behavior. An example of the task is shown in Figure 1B. In this task, participants controlled the right arm of a virtual character using the up and down arrow keys to adjust the shooting angle and used the spacebar to fire at the balloon target. Two visual prompt conditions were included: (1) static cue prompts, in which a fixed target marker was presented in advance at the location where the balloon would subsequently appear; and (2) dynamic combined prompts, in which a target marker gradually disappeared in a clockwise direction at the upcoming target location, followed immediately by the appearance of a balloon at the same location after the marker completely disappeared.

Both prompt conditions were presented within the same TGT session. The formal TGT consisted of one block containing 40 trials, including 20 static cue prompt trials and 20 dynamic combined prompt trials. The two prompt conditions were randomly intermixed on a trial-by-trial basis rather than being presented in separate blocks. Each trial contained only one prompt condition, and the order of static and dynamic combined prompt trials was randomized for each participant. This randomized presentation design was adopted to minimize potential order effects when comparing the two prompt conditions.

Each TGT trial began with a 3 s visual prompt phase. During the prompt phase, either a static cue prompt or a dynamic combined prompt was presented according to the randomized trial condition. The balloon target was generated at a random height directly in front of the virtual character’s right arm. Participants adjusted the shooting angle using the up and down arrow keys and pressed the spacebar to fire. After the target balloon appeared, participants used the up and down arrow keys to adjust the shooting angle and pressed the spacebar to fire. When the balloon was hit and exploded, the system judged the trial as a successful hit. Each balloon was time-limited to 60s. If the participant failed to successfully hit the target within the specified time limit, that trial was automatically terminated and recorded as a miss.

Reaction time was defined as the interval from balloon target onset to the firing action, that is, from the appearance of the balloon target to the participant pressing the spacebar to shoot. This measure reflected the time required for target perception, shooting-angle adjustment, and initiation of the firing response. Because the behavioral analysis primarily focused on the time efficiency of goal-directed task performance, only trials in which the target was successfully hit were included in the reaction-time analysis. Hit status was used mainly to determine whether a trial was validly completed and was not analyzed as an independent behavioral outcome.

After each trial, regardless of whether the target was successfully hit, the virtual arm was automatically reset to a predefined initial angle, and the next balloon target was generated at a new random height.

### 2.4 Data preprocessing

fNIRS data were preprocessed using the HOMER2 toolbox in combination with MATLAB R2023a. Raw light intensity signals were first converted to optical density based on the modified Beer–Lambert law. Motion artifacts caused by head movements and swallowing were then identified using a motion detection module (tMotion = 1.0 s, tMask = 1.0, STDEVthresh = 10.0, AMPthresh = 5.00), followed by correction using a wavelet-based denoising method (Wavelet transform, Daubechies 4). Afterward, a band-pass filter (0.01–0.08 Hz) was applied to remove physiological noise (e.g., cardiac artifacts) and low-frequency drift. The optical density signals were subsequently converted into concentration changes of deoxygenated hemoglobin and oxygenated hemoglobin (HbO₂). Finally, the onset of visual prompts in the virtual environment was defined as the key event time point of the TGT. Based on the time-locking principle, fNIRS data were segmented and analyzed accordingly. Block averaging was performed within the target stimulus window (−500 to 1,500 ms) to obtain event-related hemodynamic responses with an improved signal-to-noise ratio, ensuring accurate extraction of neural signal features.

### 2.5 Data analysis

To systematically investigate the mechanisms by which static cue prompts and dynamic combined prompts influence attention–motor performance, the data analysis pipeline in this study consisted of three modules: behavioral analysis, hemodynamic signal processing, and brain functional network analysis.

For the SART, raw behavioral data exported from E-Prime 3.0 were preprocessed in Excel and subsequently subjected to statistical analysis. Independent-samples t-tests were conducted to compare sustained attention performance between the stroke group and the healthy control group. The evaluation metrics included reaction time, coefficient of variation of reaction time (standard deviation/mean), number of commission errors (responses to the No-go stimulus, digit 3), and number of omission errors (failures to respond to Go stimuli). For the TGT, reaction time data, defined as the interval between balloon target onset and the firing action (i.e., the spacebar press), were recorded in real time using the Unity engine. A two-way repeated-measures analysis of variance (ANOVA) was conducted to examine differences in TGT reaction time between groups and visual prompt conditions (static cue prompts vs. dynamic combined prompts).

Hemodynamic signal processing was performed using a baseline correction method. The onset of visual prompts was defined as time zero, and the 2-s period immediately preceding this time point was used to establish the resting baseline. Signal normalization was achieved by subtracting the baseline mean from the oxygenated hemoglobin (HbO₂) concentration. Given that HbO₂ is more sensitive to task-related cortical hemodynamic changes, it was selected as the primary hemodynamic indicator for analysis.

Following visual prompt onset, HbO₂ concentration changes were examined over a 0–20 s period, and the mean HbO₂ concentration within the 0–10 s window was calculated for statistical analysis. The 0–10 s window was defined as the primary analysis window for task-evoked HbO₂ responses based on the temporal characteristics of the fNIRS hemodynamic response. Previous studies have shown that task-evoked HbO₂ changes typically emerge several seconds after stimulus onset and reach a major response phase at approximately 5–6 s after stimulation (32). In addition, inspection of the HbO₂ time courses in the present study showed apparent changes approximately 5–10 s after prompt onset in both groups (Figures 2A,B). Therefore, the 0–10 s window was considered appropriate for characterizing the early hemodynamic response evoked by the visual prompts.

**Figure 2:**
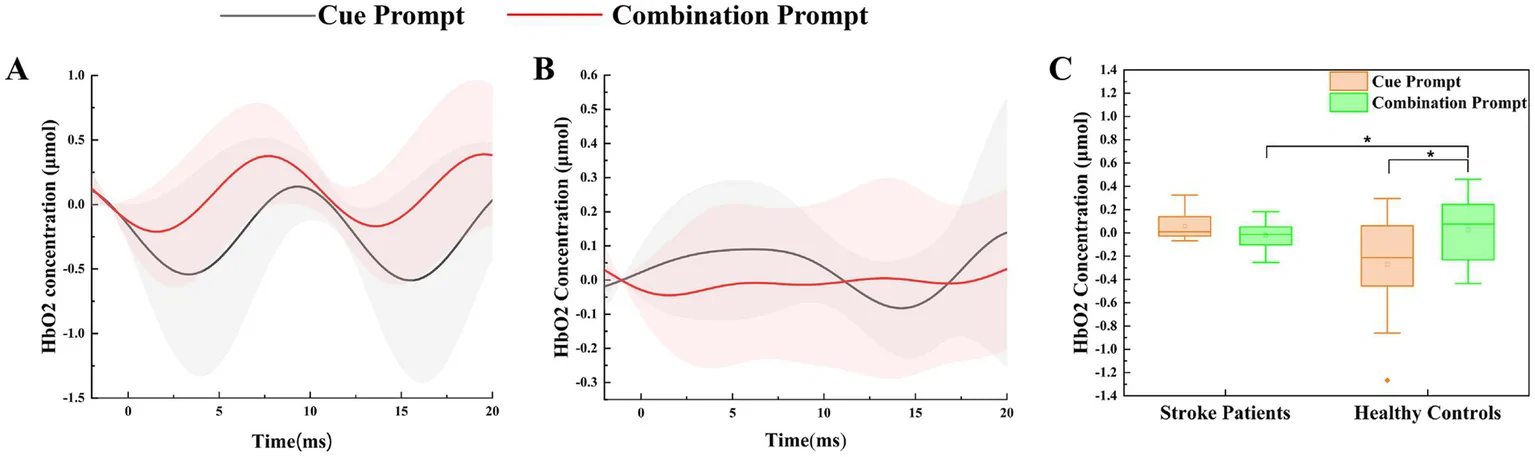
Analysis results of cerebral hemodynamic measures, specifically HbO_2_ concentration. **(A)** The HbO_2_ concentration in healthy subjects under different prompt conditions, with colored lines representing distinct prompt types. **(B)** The HbO_2_ concentration in stroke patients under different prompt conditions, with colored lines representing distinct prompt types. **(C)** Statistical analysis results for the two groups of subjects. Different colors distinguish between prompt types. Horizontal lines represent the median, hollow rectangles represent the mean, and solid rhombuses indicate outliers. *p < 0.05, **p < 0.01, ***p ≤ 0.001.

Brain functional networks were constructed based on Pearson correlation coefficients, which are widely used to characterize functional connectivity between fNIRS channels (33). Unlike the HbO₂ concentration analysis, which focused on the magnitude of the early hemodynamic response, functional connectivity analysis requires a relatively longer time series to estimate temporal correlations between channels. Therefore, HbO₂ data from the 0–20 s period following visual prompt onset were used for functional connectivity analysis. For each participant and experimental condition, Pearson correlation coefficients were calculated between the HbO₂ time series of all channel pairs within this period, resulting in a 46 × 46 weighted functional connectivity matrix. The Pearson correlation coefficient was calculated as follows:

$$r_{\mathrm{XY}}=\frac{\sum_{i=1}^{n}(X_{i}-\overset{-}{X})(Y_{i}-\overset{-}{Y})}{\sqrt{\sum_{i=1}^{n}{\left(X_{i}-\overset{-}{X}\right)}^{2}}\sqrt{\sum_{i=1}^{n}{\left(Y_{i}-\overset{-}{Y}\right)}^{2}}}$$

Where $X_{i}$ and $Y_{i}$ represent the HbO₂ concentration values of channels X and Y at time point t, $\overset{-}{X}$ and $\overset{-}{Y}$ denote the mean values of the corresponding time series, and n is the total number of time points.

Negative Pearson correlation coefficients were set to zero, and only positive correlations were retained for subsequent network construction and graph-theoretical analysis. This approach was adopted because the present study focused on functional connectivity reflecting positive synchronization of hemodynamic activity across cortical regions. In fNIRS signals, positive correlations indicate covarying HbO₂ fluctuations between regions, whereas the physiological interpretation of negative correlations is less straightforward (34). In addition, negative correlations may be influenced by systemic physiological fluctuations, measurement noise, and preprocessing procedures (35). Therefore, only positive correlations were retained to ensure a more consistent interpretation of functional connections across participants and experimental conditions. The definition of the positive connectivity network was also consistent with the conventional graph-theoretical methods used in this study to calculate network topology measures.

The resulting positive-weight functional connectivity matrix was subsequently subjected to Fisher r-to-z transformation to improve the distributional properties of the correlation coefficients. A sparsity-based thresholding approach was then applied to the transformed connectivity matrix. The sparsity range was set from 0.1 to 0.5, with an increment of 0.1. At each sparsity level, connections with the highest weights were retained until the predefined network density was reached, and the retained connections were assigned a value of 1, whereas the remaining connections were assigned a value of 0. This procedure generated a binary adjacency matrix at each sparsity level.

$$Z_{\mathrm{XY}}=\frac{1}{2}\ln(\frac{1+r_{\mathrm{XY}}}{1-r_{\mathrm{XY}}})$$

The use of a fixed sparsity threshold ensured that the resulting networks had the same edge density across participants and experimental conditions, thereby improving the comparability of graph-theoretical measures (36). The clustering coefficient, characteristic path length, and small-worldness were subsequently calculated from the resulting binary networks using the Brain Connectivity Toolbox (34). All graph-theoretical measures were calculated separately at each sparsity threshold.

### 2.6 Statistical analysis

All statistical analyses were performed using SPSS version 22.0. Normality of continuous variables was first assessed using the Shapiro–Wilk test, and homogeneity of variance was examined using Levene’s test to ensure the applicability of parametric analyses. For the SART, independent-samples t-tests were conducted to compare stroke patients and healthy controls in terms of reaction time, coefficient of variation of reaction time, number of commission errors, and number of omission errors. For the TGT, a 2 (group: stroke patients vs. healthy controls) × 2 (prompt type: static cue prompts vs. dynamic combined prompts) repeated-measures analysis of covariance (ANCOVA) was performed to examine the effects of group and prompt type on reaction time and prefrontal HbO₂ concentration. In this model, prompt type was treated as a within-subject factor, and group was treated as a between-subject factor. For significant main effects or interaction effects, simple effects analysis or *post hoc* multiple comparisons were further conducted. Bonferroni correction was applied to all *post hoc* comparisons to control for the inflation of Type I error due to multiple testing. For graph-theoretical analysis, network metrics were calculated across five sparsity thresholds (0.1 to 0.5, step = 0.1), followed by a 2 × 2 repeated-measures ANCOVA. To address the issue of multiple comparisons, the Benjamini–Hochberg false discovery rate (FDR) method was applied to correct the *p*-values for the main effects of group, prompt type, and their interaction. In all analyses, a *p*-value < 0.05 was considered statistically significant.

## 3 Results

### 3.1 Behavioral performance

To compare behavioral performance on the SART between the two groups, independent-samples t-tests were conducted on the SART behavioral measures. The results (Table 1) showed that the mean reaction time in the healthy control group (303.75 ± 30.30 ms) was significantly faster than that in the stroke group (537.31 ± 166.18 ms), with a statistically significant difference (t = 4.79, *p* < 0.001). The coefficient of variation of reaction time in the healthy control group (0.19 ± 0.05) was significantly lower than that in the stroke group (0.39 ± 0.18) (t = 3.60, *p* < 0.001). Regarding omission errors, the healthy control group (15.08 ± 7.49) made fewer omissions than the stroke group (32.08 ± 38.44); however, this difference did not reach statistical significance (t = 1.50, *p* = 0.116). The standard deviation of omission errors was 38.44 in the stroke group. Similarly, for commission errors, the healthy control group (2.75 ± 1.54) showed fewer errors than the stroke group (4.83 ± 4.37), although the difference was not statistically significant (t = 1.56, *p* = 0.104). These statistical results are illustrated using boxplots in Figure 3A.

**Table 1:** Comparison of behavioral indicators of the sustained attention task.

Indicators	Stroke group (*n* = 12)	Healthy controls (*n* = 12)	*t*	*p*
Reaction time	537.31 ± 166.18	303.75 ± 30.30	4.79	<0.001
Reaction time variability	0.39 ± 0.18	0.19 ± 0.05	3.60	<0.001
Number of omissions	32.08 ± 38.44	15.08 ± 7.49	1.50	0.116
Error count	4.83 ± 4.37	2.75 ± 1.54	1.56	0.104

**Figure 3:**
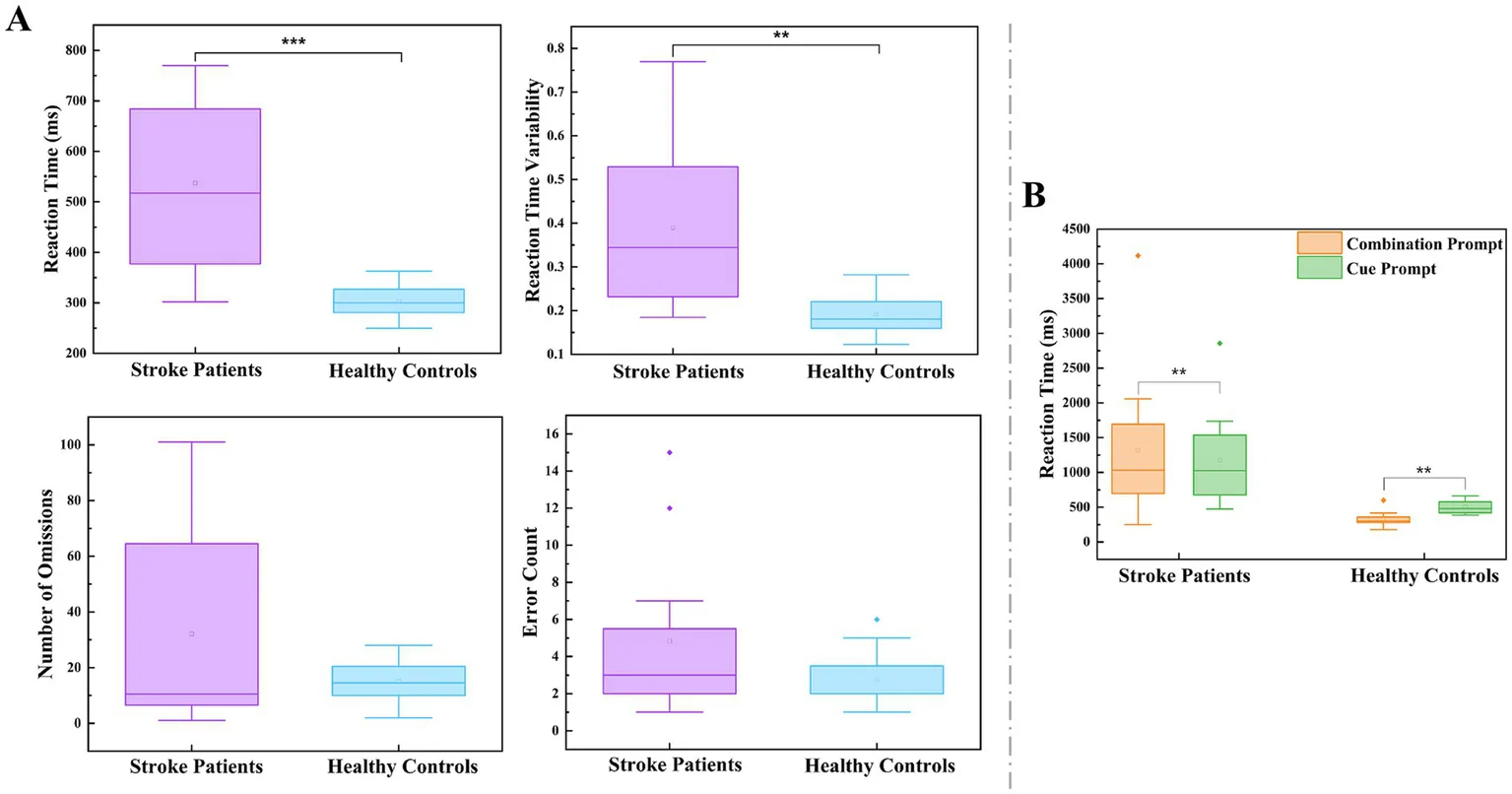
Behavioral statistical analysis results of stroke patients and healthy subjects in continuous attention tasks and target-guided tasks. **(A)** Statistical analysis results of behavioral performance in the sustained attention task for stroke patients and healthy subjects, including reaction time, reaction time variability, number of missed responses, and number of incorrect responses. Different colors represent different groups. Horizontal lines indicate the median, hollow rectangles represent the mean, and solid rhombuses mark outliers. *p < 0.05, **p < 0.01, *** *p* ≤ 0.001. **(B)** The statistical analysis of behavioral performance, specifically reaction time, in target-guided tasks for stroke patients and healthy subjects shows that different colors distinguish between prompt types. Horizontal lines represent the median, hollow rectangles represent the mean, and solid rhombuses indicate outliers. *p < 0.05, **p < 0.01, ***p ≤ 0.001.

To investigate the effects of different types of visual prompts on attention-motor performance in the two groups, a 2 (group: stroke patients vs. healthy controls) × 2 (prompt type: static cue prompts vs. dynamic combined prompts) repeated-measures analysis of covariance (ANCOVA) was conducted, with reaction time in the TGT as the dependent variable and centered age (age_c) included as a covariate. Prior to the formal analysis, the homogeneity of regression slopes assumption was tested. The results showed that the interaction between group and age was not significant under either the static cue prompt condition (*p* = 0.565) or the dynamic combined prompt condition (*p* = 0.598), satisfying the assumptions for ANCOVA. The analysis results (Table 2, Figure 3B) revealed a significant interaction between group and prompt type (*F* = 4.62, *p* = 0.042, η^2^ = 0.181).

**Table 2:** Reaction time and covariance analysis results for target-goal tasks.

Comparison type	F	*p*	η^2^
Group	81.19	<0.001	0.794
Prompt Type	2.28	0.146	0.098
Age (covariate)	6.05	0.023	0.224
Interaction			
Group × Prompt Type	4.62	0.042	0.181
Prompt Type × Age	1.63	0.216	0.072

Simple effects analysis with Bonferroni correction (Table 3) further showed that, in the healthy control group, reaction time under the dynamic combined prompts condition (adjusted mean: 341.2 ± 21.7 ms) was significantly faster than that under the static cue prompts condition (492.8 ± 26.4 ms), with a mean difference of 151.6 ms (*p* = 0.003). In contrast, in the stroke group, reaction time under the static cue prompts condition (adjusted mean: 1158.4 ± 98.3 ms) was significantly faster than that under the dynamic combined prompts condition (1342.7 ± 112.5 ms), with a mean difference of −184.3 ms (*p* = 0.018). Between-group comparisons showed that reaction times in the healthy control group were significantly faster than those in the stroke group under both prompt conditions: under the static cue prompts condition (492.8 ms vs. 1158.4 ms), the mean difference was 665.6 ms (*p* < 0.001); under the dynamic combined prompts condition (341.2 ms vs. 1342.7 ms), the mean difference was 1001.5 ms (*p* < 0.001). Covariate analysis revealed a significant main effect of age on reaction time (*F* = 6.05, *p* = 0.023, η^2^ = 0.224).

**Table 3:** Estimated marginal means of reaction times controlling for age.

Group	Static cue prompts (ms)	Dynamic combined prompts (ms)	Within-group comparison (*p*)	Effect direction
Healthy controls	492.8 ± 26.4	341.2 ± 21.7	0.003	Dynamic > Static
Stroke group	1158.4 ± 98.3	1342.7 ± 112.5	0.018	Static > Dynamic
Between-group comparison	*p* < 0.001	*p* < 0.001		

### 3.2 Cerebral hemodynamic response

Figures 2A,B present the averaged time courses of prefrontal HbO₂ concentration in the healthy control group and stroke patients under different prompt conditions, respectively, with shaded areas representing the error bands. Time zero was defined as the onset of the visual prompt, and the interval from −2 to 0 s was used as the baseline period. It was observed that following the presentation of the visual prompt, HbO₂ concentration gradually increased and reached the first peak between 5 and 10 s, followed by a second peak after the execution of the motor response. Therefore, the mean HbO₂ concentration within the 0–10 s time window was extracted for subsequent analysis.

To investigate the effects of different types of visual prompts on prefrontal cortical activation, a 2 (group: stroke patients vs. healthy controls) × 2 (prompt type: static cue prompts vs. dynamic combined prompts) repeated-measures analysis of covariance (ANCOVA) was conducted, with the mean HbO₂ concentration within the 0–10 s time window during the TGT as the dependent variable and centered age (age_c) included as a covariate. Prior to the formal analysis, the homogeneity of regression slopes assumption was tested. The results showed that the interaction between group and age was not significant under either the static cue prompts condition (*p* = 0.399) or the dynamic combined prompts condition (*p* = 0.356), satisfying the assumptions for ANCOVA. The ANCOVA results (Table 4) revealed a significant main effect of group (*p* = 0.027), whereas the main effect of prompt type was not significant (*p* = 0.356). Importantly, a significant interaction between group and prompt type was observed (*F* = 6.62, *p* = 0.018, η^2^ = 0.240).

**Table 4:** Two-factor repeated measures ANCOVA results for prefrontal HbO_2_ concentration.

Comparison type	F	*p*	η^2^
Group	5.68	0.027	0.213
Prompt type	0.89	0.356	0.041
Age (covariate)	4.78	0.040	0.185
Interaction			
Group × Prompt Type	6.62	0.018	0.240
Prompt Type × Age	1.89	0.184	0.083

Further simple effects analysis with Bonferroni correction (Table 5) showed that, in the healthy control group, HbO₂ concentration under the dynamic combined prompts condition (adjusted mean: 0.0336 ± 0.0392) was significantly higher than that under the static cue prompts condition (−0.2418 ± 0.0348), with a mean difference of 0.2754 (*p* < 0.001). In contrast, in the stroke group, the difference in HbO₂ concentration between the two prompt conditions was not statistically significant; however, HbO₂ concentration under the static cue prompts condition (0.0612 ± 0.0348) was higher than that under the dynamic combined prompts condition (−0.0167 ± 0.0392) (*p* = 0.142). Between-group comparisons indicated that, under the static cue prompts condition, HbO₂ concentration in the stroke group was significantly higher than that in the healthy control group (mean difference = 0.3030, *p* < 0.001), whereas no significant difference was observed between groups under the dynamic combined prompts condition (*p* = 0.374). Statistical results are summarized in Figure 2C. Covariate analysis revealed a significant main effect of age on HbO₂ concentration (*F* = 4.78, *p* = 0.040, η^2^ = 0.185).

**Table 5:** Estimated marginal mean values of prefrontal HbO_2_ concentrations after controlling for age.

Group	Static cue prompts	Dynamic combined prompts	*p*	Effect direction
Stroke group	0.0612 ± 0.0348	−0.0167 ± 0.0392	0.142	No significant difference
Healthy controls	−0.2418 ± 0.0348	0.0336 ± 0.0392	<0.001	Static < Dynamic
Between-group comparison	p < 0.001	*p* = 0.374		

### 3.3 Functional brain network properties

Figures 4A,B present the functional connectivity adjacency matrices of the stroke and healthy control groups under the two prompt conditions. Figures 5A,B display the graph-theoretical metrics of brain functional networks, including clustering coefficient (CC), characteristic path length (PL), and small-worldness, in stroke patients and healthy controls under different prompt conditions, with shaded areas representing within-group variability (error bands). Under both prompt conditions, brain functional networks in both groups exhibited small-world properties (small-worldness > 1).

**Figure 4:**
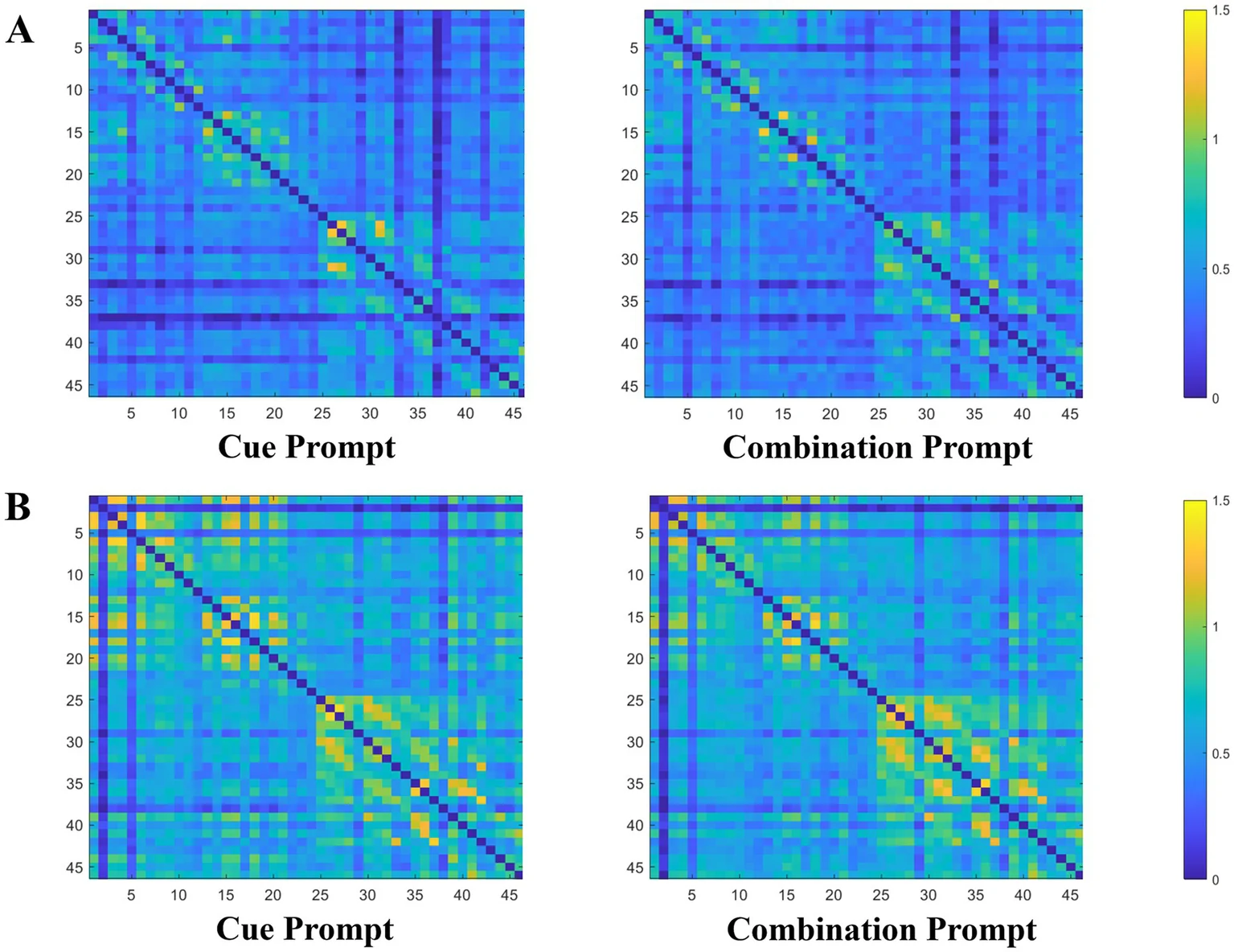
The adjacency matrix diagram of brain functional connectivity. **(A)** The adjacency matrix diagram of brain functional connectivity in stroke patients. **(B)** The adjacency matrix diagram of brain functional connectivity in healthy subjects.

**Figure 5:**
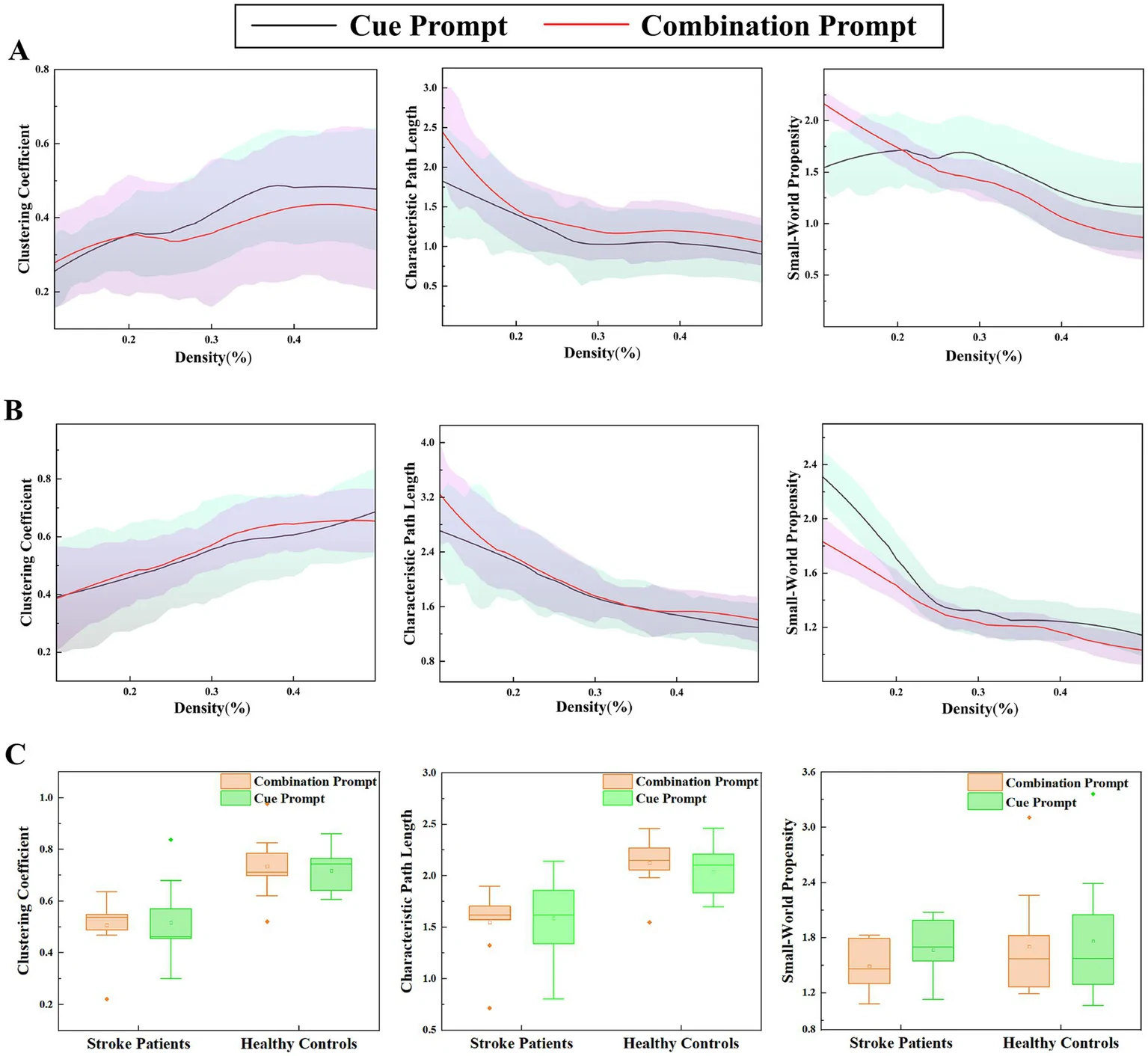
Graph theory indicators and statistical analysis results of brain networks. **(A)** Graph theory indicators for stroke patients. **(B)** Graph theory indicators of healthy subjects. **(C)** Statistical analysis results for stroke patients and healthy subjects. Different colors distinguish between prompt conditions. Horizontal lines represent the median, hollow rectangles represent the mean, and solid rhombuses indicate outliers. *p < 0.05, **p < 0.01, ***p ≤ 0.001.

To further examine the effects of different visual prompts on the topological properties of brain functional networks, a 2 (group) × 2 (prompt type) repeated-measures analysis of covariance (ANCOVA) was conducted on CC and PL at each sparsity threshold (0.1–0.5), with age included as a covariate. The false discovery rate (FDR) method was applied to correct for multiple comparisons.

The results (Table 6) showed that the main effect of group on CC was significant at thresholds from 0.1 to 0.4 (FDR-corrected *q* < 0.05) and marginally significant at the threshold of 0.5 (*p* = 0.051, *q* = 0.051). At all thresholds, CC in the healthy control group was significantly higher than that in the stroke group (all between-group comparisons *p* < 0.01). Neither the main effect of prompt type nor the group × prompt type interaction reached significance at any threshold (q > 0.05). For PL, the main effect of group was significant at thresholds ranging from 0.1 to 0.3 (q < 0.05), but not at thresholds of 0.4 and 0.5 (q > 0.05). At all thresholds, PL in the healthy control group was significantly longer than that in the stroke group (all between-group comparisons *p* < 0.01). Similarly, neither the main effect of prompt type nor the interaction effect was significant (q > 0.05). Covariate analysis revealed that age did not show a significant main effect at any threshold (CC: *F* = 0.31–1.18, all *p* > 0.05; PL: *F* = 0.72–1.45, all *p* > 0.05).

**Table 6:** Results of repeated measures covariance analysis of graph theory indices under different density thresholds.

Density threshold	Type of effect	CC			PL		
		F	*p*	Q(FDR)	F	*p*	Q(FDR)
0.1	Group effect	12.84	0.002	0.010	8.27	0.011	0.028
	Prompt type effect	0.92	0.351	0.351	0.38	0.546	0.546
	Interactions	5.96	0.026	0.065	2.95	0.105	0.263
0.2	Group effect	10.73	0.005	0.013	6.91	0.018	0.030
	Prompt type effect	1.21	0.288	0.360	0.73	0.405	0.506
	Interactions	4.85	0.042	0.070	2.21	0.156	0.260
0.3	Group effect	8.91	0.009	0.015	5.34	0.034	0.043
	Prompt Type Effect	1.54	0.232	0.290	1.08	0.314	0.393
	Interactions	3.62	0.074	0.093	1.62	0.221	0.276
0.4	Group effect	6.78	0.019	0.024	3.82	0.068	0.085
	Prompt type effect	1.83	0.195	0.244	1.41	0.252	0.315
	Interactions	2.51	0.133	0.166	1.09	0.312	0.347
0.5	Group effect	4.42	0.051	0.051	2.76	0.116	0.116
	Prompt type effect	2.09	0.167	0.209	1.73	0.207	0.259
	Interactions	1.76	0.203	0.203	0.82	0.378	0.378

To verify the overall trend, further repeated-measures ANCOVA was conducted on CC, PL, and small-worldness across the full sparsity range (0.1–0.5). The results showed a significant main effect of group for both CC (*F* = 58.27, *p* < 0.001, η^2^ = 0.785) and PL (*F* = 36.41, *p* < 0.001, η^2^ = 0.695), whereas neither the main effect of prompt type nor the interaction effect was significant (*p* > 0.05). No significant main effects of group (*p* = 0.343) or prompt type (*p* = 0.435) were observed for small-worldness. A summary of these results is presented in Figure 5C.

## 4 Discussion

### 4.1 Core findings

The present findings indicate that the behavioral and hemodynamic responses to visual prompts differed between stroke patients and healthy controls. Specifically, healthy controls showed faster responses under the dynamic combined prompt condition, whereas stroke patients showed faster responses under the static cue prompt condition. The HbO₂ responses also differed between the two groups across the two visual prompt conditions. Age as a covariate was significantly associated with TGT reaction time and HbO₂ concentration. Meanwhile, the group × prompt type interaction remained significant after age was included in the statistical model, indicating that the observed differences in prompt-related effects between the groups cannot be fully explained by age differences. Although age differed substantially between the groups, these findings suggest that age may have influenced the study outcomes but does not fully account for the observed group-dependent differences in responses to the visual prompt conditions.

Importantly, Behavioral performance and HbO₂ responses both showed significant group × prompt type interactions, indicating different response patterns to the visual prompt conditions between the two groups. In contrast, the brain network measures primarily showed group differences, with no significant main effect of prompt type or group × prompt type interaction. These findings suggest that, unlike the behavioral and hemodynamic measures, the network-level measures did not provide evidence of prompt-dependent changes in network topology.

### 4.2 Behavioral mechanism

At the behavioral level, stroke patients exhibited slower reaction times and greater variability in the SART. In the TGT, the significant group × prompt type interaction further revealed that dynamic combined prompts improved performance in healthy individuals but impaired performance in stroke patients.

The dissociation in behavioral outcomes may be associated with differences in sustained attention between the two groups. Differences in sustained attention may be associated with the different response patterns to visual prompts observed between the two groups; however, the present study did not directly examine the associations between SART performance and TGT performance or neurophysiological measures. Therefore, this interpretation remains hypothetical. Although dynamic combined prompts integrate both spatial and temporal predictive information and may provide additional support for motor preparation when sufficient attentional resources are available, the present findings cannot determine whether the additional processing demands imposed by dynamic information exceeded the attentional capacity of stroke patients. Future studies with larger samples and direct association analyses are required to further clarify the role of sustained attention in modulating responses to different visual prompt strategies.

### 4.3 Hemodynamic mechanism

At the neural level, the interaction effect observed in prefrontal HbO₂ concentration provided additional physiological evidence for the aforementioned behavioral dissociation. Healthy controls exhibited higher HbO₂ levels under the dynamic combined prompts condition, whereas stroke patients did not show significant differences between prompt conditions. This pattern suggests that dynamic cues may be more effective in recruiting prefrontal resources related to attentional regulation and motor preparation when attentional resources are sufficient.

### 4.4 Network-level interpretation

At the network level, significant group differences in clustering coefficient and characteristic path length indicated differences in functional network organization between stroke patients and healthy controls. The higher clustering coefficient and shorter characteristic path length observed in healthy controls suggest relatively more efficient functional network organization, whereas the corresponding network properties in stroke patients may reflect alterations in functional network organization associated with post-stroke changes.

Importantly, neither the main effect of prompt type nor the group × prompt type interaction reached statistical significance at the network level. Therefore, the network findings should be interpreted primarily as reflecting stroke-related group differences rather than dynamic changes in network topology induced by different visual prompt conditions. Accordingly, the present results do not provide evidence that the different visual prompt conditions directly modulated network topology.

### 4.5 Boundary conditions and limitations

Several limitations should be considered when interpreting the present findings. First, although age was statistically controlled using ANCOVA, the substantial age difference between groups remains a critical limitation. ANCOVA may not fully account for age-related differences in neurovascular coupling. Future studies should adopt age-matched designs to minimize the potential influence of age differences on behavioral and neurophysiological outcomes and to improve the validity of between-group comparisons.

Second, the sample size was relatively small (*n* = 12 per group), which limits the generalizability and statistical stability of the findings. Although the behavioral interaction showed a relatively large effect size, the hemodynamic measures exhibited greater variability, and the present findings should therefore be considered preliminary.

Third, although all patients were in the chronic and clinically stable phase, heterogeneity in lesion characteristics and clinical severity may have influenced both behavioral and neural outcomes. Future studies should incorporate more detailed clinical assessments.

Fourth, the use of different hands between groups (non-paretic side in patients vs. right hand in healthy controls) may have affected reaction time measures. Future research should control for hand dominance or optimize the experimental paradigm to minimize this potential confound.

In addition, the present study did not further examine the associations between SART performance and TGT behavioral performance, HbO₂ responses, or brain network measures. Therefore, the current findings cannot determine whether sustained attention performance is directly associated with differences in behavioral and neurophysiological responses under different visual prompt conditions. Future studies should recruit larger samples and use preregistered correlation analyses, multiple regression analyses, or other appropriate statistical approaches to further examine the relationship between sustained attention performance and the effects of visual prompts.

### 4.6 Implications

The present study provides preliminary evidence that different visual prompt strategies are associated with distinct behavioral and neurophysiological response patterns in individuals with stroke. By combining behavioral measures, fNIRS-based hemodynamic responses, and functional brain network analysis, this study contributes to a better understanding of the differences in behavioral and neural responses associated with different visual prompt conditions after stroke. Different sustained attention states may be related to the different response patterns observed between the two groups; however, the present study did not directly examine the associations between SART performance and TGT performance or neurophysiological measures. Therefore, this interpretation remains hypothetical and requires further empirical verification.

Although the present findings do not directly demonstrate improvements in rehabilitation outcomes, motor learning, or functional recovery, they provide potential insights for future investigations of adaptive visual prompt strategies in virtual reality (VR)-based rehabilitation environments. Future longitudinal intervention studies are needed to determine whether individualized visual prompt systems can effectively support rehabilitation training and produce meaningful functional benefits.

## 5 Conclusion

In conclusion, this study demonstrated that static cue prompts and dynamic combined prompts elicited different behavioral and neurophysiological response patterns in stroke patients and healthy controls. Specifically, dynamic combined prompts facilitated faster responses and enhanced prefrontal activation in healthy controls, whereas stroke patients showed better task performance under the static cue prompt condition. Further analyses indicated that dynamic combined prompts were associated with distinct task-related behavioral patterns and changes in cortical hemodynamic activity, while functional brain network measures primarily reflected group differences between stroke patients and healthy controls rather than prompt-dependent changes in network topology. These findings suggest that the effects of different visual prompt strategies may vary according to individual characteristics. Although differences in sustained attention capacity may be related to the distinct response patterns observed between the two groups under the two visual prompt conditions, the present study did not directly examine the associations between SART performance and TGT performance, HbO₂ responses, or functional network measures. Therefore, the role of sustained attention in the observed visual prompt effects remains hypothetical.

These findings provide preliminary behavioral and neurophysiological evidence for understanding the differential responses of stroke patients and healthy individuals to different visual prompt strategies, and further suggest that visual prompt design may need to take into account individual differences in attentional function and information-processing capacity. However, whether such prompt strategies can further facilitate rehabilitation training or improve functional recovery remains to be determined. Future studies should include larger samples and use prespecified association analyses, such as correlation or multiple regression analyses, to further examine the relationship between sustained attention performance and visual prompt effects. In addition, longitudinal intervention designs and clinically relevant rehabilitation outcomes are needed to evaluate the practical value of individualized and adaptive visual prompt strategies in stroke rehabilitation.

## Data Availability

The raw data supporting the conclusions of this article will be made available by the authors, without undue reservation.
